# Proteomic Analyses of Vitreous in Proliferative Diabetic Retinopathy: Prior Studies and Future Outlook

**DOI:** 10.3390/jcm10112309

**Published:** 2021-05-25

**Authors:** Sarah R. Weber, Yuanjun Zhao, Christopher Gates, Jingqun Ma, Felipe da Veiga Leprevost, Venkatesha Basrur, Alexey I. Nesvizhskii, Thomas W. Gardner, Jeffrey M. Sundstrom

**Affiliations:** 1 Department of Ophthalmology, Penn State College of Medicine, 500 University Drive, Hershey, PA 17033, USA; sweber2@pennstatehealth.psu.edu (S.R.W.); yuz14@psu.edu (Y.Z.); 2 Kellogg Eye Center, University of Michigan Medical School, 1000 Wall Street, Ann Arbor, MI 48105, USA; tomwgard@med.umich.edu; 3 Bioinformatics Core, Biomedical Research Core Facilities, University of Michigan Medical School, 2800 Plymouth Road, Ann Arbor, MI 48109, USA; cgates@med.umich.edu; 4 Department of Pathology, St. Jude Children’s Research Hospital, 262 Danny Thomas Place, Memphis, TN 38105, USA; majingqun@gmail.com; 5 Department of Pathology, University of Michigan Medical School, 1301 Catherine Street, Ann Arbor, MI 48109, USA; felipevl@umich.edu (F.d.V.L.); vbasrur@med.umich.edu (V.B.); nesvi@med.umich.edu (A.I.N.); 6 Department of Computational Medicine and Bioinformatics, University of Michigan, 100 Washtenaw Ave, Ann Arbor, MI 48109, USA

**Keywords:** mass spectrometry, proteomics, bioinformatics, vitreous, proliferative diabetic retinopathy

## Abstract

Vitreous fluid is becoming an increasingly popular medium for the study of retinal disease. Numerous studies have demonstrated that proteomic analysis of the vitreous from patients with proliferative diabetic retinopathy yields valuable molecular information regarding known and novel proteins and pathways involved in this disease. However, there is no standardized methodology for vitreous proteomic studies. Here, we share a suggested protocol for such studies and outline the various experimental and analytic methods that are currently available. We also review prior mass spectrometry-based proteomic studies of the vitreous from patients with proliferative diabetic retinopathy, discuss common pitfalls of these studies, and propose next steps for moving the field forward.

## 1. Introduction

The vitreous is a transparent, minimally cellular extracellular matrix that fills the posterior cavity of the eye and abuts the retina. It consists mainly of water (98%). The remaining 2% consist of proteins, extracellular matrix components, and other compounds [1]. The major protein component is collagen, with types II and IX predominating [2,3]. Glycosaminoglycans, including hyaluronan and chondroitin sulfate, are also major components. Plasma proteins, including hemoglobin, albumin, transthyretin, and others are also abundantly present in a normal human vitreous [4]. Though historically considered protein-poor, recent experiments have identified between 1000 and 2500 unique proteins in the human vitreous as detailed in later sections.

Within the complex vitreous proteome, a substantial number of proteins with well-established roles in retinal physiology and disease have been identified. A recent paper demonstrated that a normal vitreous contains more than 40 proteins known to play major roles in retinal disease [5]. Analyses of the vitreous derived from patients with various retinal conditions, including diabetic retinopathy (reviewed here and in [6,7]), retinal vein occlusion [8,9], retinal detachment [10,11], retinoblastoma [12], age-related macular degeneration [13,14], retinopathy of prematurity [15], etc., have uncovered proteins and pathways relevant to the known diagnoses, as well as, in some cases, novel protein components that may uncover new disease mechanisms. As such, the vitreous is able to serve as a proximal biofluid of the retina and is an ideal tissue for molecular interrogation in the setting of retinal disease.

Due to the complexity of the vitreous protein composition, mass spectrometry (MS)-based proteomics has become the preferred method for its molecular analysis, as it is able to uncover more information than targeted methods such as enzyme-linked immunosorbent assay (ELISA) or western blotting. Further, because proteins are the effectors and regulators of essentially all biological processes, proteomics has an advantage over other ‘-omics’ fields in that its focus is the biomolecules most directly involved in functional processes. Unbiased proteomic methods, which aim to uncover the entire proteome of a given sample, facilitate discovery of novel proteins and pathways that may be relevant to the tissue or disease of interest. In this way, proteomic analysis of the vitreous humor has already begun to advance the field of ophthalmology, with the majority of proteomic studies focusing on enhancing the understanding of proliferative diabetic retinopathy (PDR). In this review, we outline the proteomic methods relevant to analyzing the vitreous, review prior MS-based proteomic studies of the PDR vitreous, and discuss opportunities for moving the field forward.

## 2. Workflow for Vitreous Proteomics

Proteomic studies of the vitreous from humans with PDR have been carried out by many different laboratories using a wide variety of workflows. These studies have varied considerably in terms of the methods utilized and the number of unique proteins identified, with some studies identifying fewer than 60 proteins and others exceeding 2400 (Table 1). Here, we provide an overview of a generalized workflow for proteomic analysis of a human PDR vitreous and outline the key parameters to consider in order to generate high-integrity datasets with a sufficient depth of coverage.

### 2.1. Sample Source and Selection

Protocols have been established for acquisition of the human vitreous in the clinical setting [16,17], from donor eyes [18], and in the operating room at the start of a pars plana vitrectomy [19]. Vitreous samples can be safely obtained in clinic with a syringe and a 25- or 26-gauge needle prior to an intravitreal medication injection [16,17], but this method has several disadvantages; namely, there is a risk, albeit very low, of retinal tear or detachment, and the biopsy attempt may be unsuccessful. In general, this method yields smaller volumes (approximately 50–200 µL) than can be obtained via pars plana vitrectomy (250–500 µL). Postmortem vitreous samples (or those from enucleated eyes from living patients) can be obtained, and the vitreous should be harvested via a syringe prior to sectioning or further dissection to ensure the desired substructure is obtained and prevent loss of material [6]. Several concerns arise when analyzing a postmortem vitreous. Retinal ischemia following death may influence the protein composition of the vitreous, and these changes may be difficult to distinguish from the proteome characteristics that were present prior to death. Our laboratory has also found that a postmortem vitreous contains extracellular vesicles (EVs) at concentrations more than 27 times those of the samples obtained in the operating room [5], further suggesting a death artifact. Additionally, variations in the amount of time between donor death and harvesting of the sample may confer differing degrees of ischemia or sample degradation. A vitreous sample obtained at the start of a pars plana vitrectomy is the most widely used sample type in proteomic studies of the PDR vitreous. In this method, undiluted fluid is obtained from the vitreous core and generally yields volumes of approximately 500 µL. In addition to practical considerations, investigators should be aware of the vitreous anatomy. Its anatomical regions consist of (1) the vitreous core or central vitreous, which comprises the majority of the total volume and contains the canal of Cloquet [20]; (2) the vitreous cortex, which surrounds the vitreous core but contains relatively more collagen in a different orientation and adheres to the retina posteriorly; (3) the vitreous base or basal vitreous, which is densely packed with collagen fibrils that affix it to the pars plana and anterior retina and prevent its removal; and (4) the anterior hyaloid in the region between the ciliary body and the lens [18,21]. These vitreous substructures are known to contain differentially expressed proteins [18]; therefore, the acquisition technique, which may determine the region(s) from which vitreous samples derive, can influence the composition of the analyzed proteome.

Detailed patient demographics are essential when planning vitreous proteomic analyses. Vitreous protein content and anatomy both change throughout development and aging, becoming increasingly liquefied with thickening fibers [22,23]. Therefore, patients’ ages and the presence or absence of a posterior vitreous detachment should be noted. Lens status also influences the structure and content of the vitreous. Altered viscosity and proteomic differences were observed in a study comparing pseudophakic versus phakic donor eyes, including reversal of the anterior–posterior viscosity gradients and differential expression of proteins including lactate dehydrogenase and transthyretin. It is not known whether these changes were associated with cataract surgery [24]. Nonetheless, information regarding lens status should be obtained. Given that the vitreous proteome changes in glaucoma [25], diabetic retinopathy (reviewed here and in [6,7]), age-related macular degeneration [13,14,26], and uveitis [27], samples from patients with any current or prior history of major ocular pathology other than the disease of interest should be excluded. The vitreous gel does not reform following its removal [21], so post-vitrectomy samples are not comparable with pre-vitrectomy samples even in the absence of other confounding variables. It is also prudent to exclude samples from patients with current or prior history of a major systemic disease, such as diabetes or cancer (except in cases where these conditions are related to the disease of interest). However, the degree to which these conditions affect the protein content of the vitreous remains unclear, as proteomic studies comparing vitreous samples with and without these confounders have not been performed.

For diabetic retinopathy (DR) samples, history of prior panretinal photocoagulation (PRP) or intravitreal anti-vascular endothelial growth factor (VEGF) injection and the time since these procedures and date of sample collection should be noted. Investigators may choose to exclude samples from patients who have recently undergone these procedures from vitreous proteomic studies, although it is unclear how these procedures affect the vitreous proteome and what timeframe cutoff is acceptable. A prior study excluded samples from patients who had undergone PRP within the six months preceding sample collection [28]. Regardless of whether such a criterion is applied, inclusion of these demographics may prove helpful for data interpretation.

A study of PDR vitreous by Hernandez et al. [28] excluded samples with hemoglobin concentration > 5 mg/mL as measured by spectrophotometry from the proteomic analysis to reduce masking of vitreous proteins with serum proteins in samples from the patients with prior vitreous hemorrhage. Whether to exclude samples based on hemoglobin concentration should be decided based on the investigator’s experimental goals, and the amount of hemoglobin one is willing to tolerate should depend on the clinical or biological question. Using a hemoglobin assay kit (Abcam, Cambridge, United Kingdom), our laboratory found that PDR samples collected from patients undergoing vitrectomy for non-clearing vitreous hemorrhage had hemoglobin concentrations ranging from undetectable in clear samples to 0.084 mg/mL (8.40 × 10^−3^ g/dL) in the samples visibly tinted red from blood (manuscript in preparation). Assuming an average hemoglobin concentration of 15 g/dL in human blood, this is 0.056% that amount. The issue of serum proteins masking more vitreous-specific proteins can be addressed with abundant protein depletion as discussed in Section 2.3.

### 2.2. Storage

Proper sample storage is critical for preservation of sample integrity. Whether the vitreous is collected in the operating room or in a clinical setting, it should be put into a sterile tube and immediately placed on wet ice. After labeling the sample with a unique identifier, it should be immediately transported to a −80 °C freezer until ready to use. Other studies suggest snap-freezing samples in liquid nitrogen prior to freezing [6], but our laboratory has found placement on wet ice to be sufficient per the validation protocol below.

### 2.3. Sample Validation and Processing

**Figure 1:**
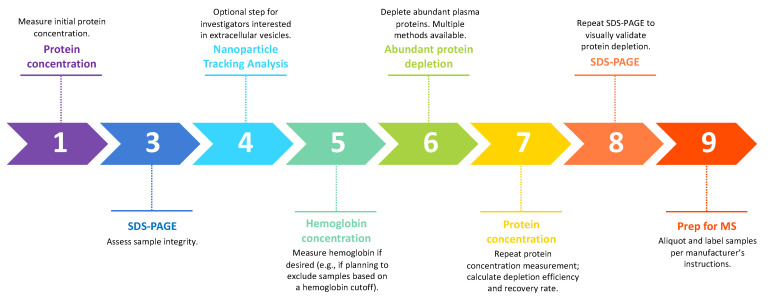
Suggested workflow for sample validation and processing. Protein concentration and sample integrity should be assessed prior to analysis. Depending on the investigators’ goals, EVs and hemoglobin may be quantified prior to further processing. Abundant proteins should be depleted and concentration should be reassessed to ensure sufficient depth of coverage. SDS-PAGE can then be repeated to validate prior steps before proceeding with MS.

Prior to preparing vitreous samples for MS, several validation and processing steps are required (Figure 1). As the first step, protein concentration should be measured. If interested in the EV-associated component of the vitreous proteome, the vitreous should first be incubated with a buffer capable of lysing EVs, such as radioimmunoprecipitation assay buffer (RIPA). To assess sample integrity, each sample should be run using sodium dodecyl sulphate-polyacrylamide gel electrophoresis (SDS-PAGE) and stained for proteins. Prominent bands are expected at 55 and 64 kDa corresponding to transthyretin and albumin, respectively. Abundant EVs were recently identified in macular hole (MH)/epiretinal membrane (ERM) and postmortem human vitreous [5]. Investigators may choose to subject samples to nanoparticle tracking analysis following SDS-PAGE. Per Section 2.1. above, hemoglobin concentration can be measured in PDR samples at this point in the processing protocol if desired. Following these optional steps, abundant protein depletion is required to facilitate wider coverage of the vitreous proteome by MS. The protein recovery rate and depletion efficiency can be calculated following a post-depletion protein concentration assay using the same method as in the first step. A recovery rate of 10–20% (i.e., a depletion efficiency of 80–90%) is expected. The samples should be again subjected to SDS-PAGE and protein staining for visual validation of adequate protein depletion. Following depletion, the bands corresponding to transthyretin and albumin should be absent from the depleted vitreous and detected in a subsequent elution fraction. Following this protocol, samples should be aliquoted into volumes appropriate for subsequent MS analysis.

#### 2.3.1. Fractionation

Fractionation of complex samples (such as the vitreous) increases proteome coverage by exposing the mass spectrometer to simpler mixtures of proteins or peptides at a given time. Fractionation can be accomplished by essentially any type of chromatography or by one- or two-dimensional electrophoresis. With electrophoretic methods, proteins are excised from the gel and separated into 20 or 40 fractions, with a higher number of fractions contributing to increased coverage. Similarly, prefractionation using chromatography can be used to separate samples into fewer or more fractions depending on the desired depth of proteome coverage. A greater number of fractions generally yields greater depth of coverage. A detailed comparison of fractionation methods can be found in [29].

#### 2.3.2. Digestion

Following fractionation, proteins are digested, traditionally with trypsin. Trypsin is the preferred enzyme as it cleaves proteins into peptides that are, on average, about ten amino acids long, an optimal length for downstream proteomic analyses. However, other proteases, such as LysC, have also been used [30].

### 2.4. Mass Spectrometry

MS is an essential tool on which diverse fields and applications rely for analysis of various types of substances, including proteins. This technique utilizes molecular or anatomic masses to elucidate the identity of an unknown compound and its components [31]. There are countless applications of MS; here, we focus on MS techniques used in bottom-up proteomic experiments to analyze biological samples.

#### 2.4.1. Mass Spectrometers: Basic Components

The three basic components of a mass spectrometer include (1) an ion source; (2) a mass analyzer; and (3) a detector. These components allow separation of gas-phase ionized components of a sample according to their mass-to-charge (m/z) ratio using electric or magnetic fields (or a combination of both). The mass spectrometer as a whole produces a mass spectrum according to signal intensity and m/z of the ions in the sample of interest where the magnitudes of peaks correspond to ion abundances [31]. There are numerous options for the various mass spectrometer components which can be combined according to the techniques and advantages that best align with experimental goals.

##### Ion Source

The two major techniques used to convert biological samples into the gas phase and ionize the constituent analytes are electrospray ionization (ESI) and matrix-assisted laser desorption/ionization (MALDI). ESI works by dissolving analytes into a liquid solvent, then transferring ions from the resultant solution into the gas phase. Because ESI relies on a liquid solvent prior to ionization, it is often coupled to separation techniques, such as liquid chromatography (for LC-MS) or gel electrophoresis, which also require samples in the liquid form [32]. Due to the large, non-volatile, and chargeable nature of proteins and other biomolecules, ESI has become exceptionally useful in biomedical proteomics experiments [31]. ESI is suitable for complex samples and is therefore an ideal ion source for proteomics experiments analyzing complex biological samples. In contrast to the liquid solvent used with ESI, MALDI generates ions from a solid crystalline matrix using pulsed laser light to evaporate and ionize the sample [31]. MALDI works best with simple peptide mixtures, and therefore is less preferred than ESI for MS of biological samples [32].

##### Mass Analyzers

The mass analyzer serves as the central component of a mass spectrometer. The following are the four most commonly used types of mass analyzers in proteomics: time-of-flight (TOF), quadrupole, ion trap, and Fourier-transform ion cyclotron (FT-MS) [32]. TOF relies on time dispersion of ions from a pulsed beam along a field-free path of predetermined length. In this technique, ion flight times vary according to m/z values, allowing for generation of mass spectra [31]. Quadrupole mass analyzers are composed of four rods, usually arranged in parallel in a square configuration. These rods exert an attractive force on ions that enter the analyzer. Ions of a particular m/z value traverse the quadrupole according to a DC voltage, radiofrequency voltage, and its frequency [31]. The resolution generated by quadrupoles is comparatively lower than that of TOF, but quadrupoles have the advantages of being simple to operate and relatively inexpensive. Ion trap mass analyzers operate similarly to quadrupoles but “trap” ions in a three-dimensional space using electric and magnetic fields rather than filtering them. In this way, the ion trap serves as an “electric-field test tube”, holding ions prior to detection. Selected ions from this “test tube” are then ejected according to their m/z value [33]. Ion trap mass analyzers have the advantages of high sensitivity and relatively low cost but have low mass accuracy [32]. The two-dimensional ion trap, however, improves upon this high sensitivity and has better mass accuracy [32,34]. FT-MS mass analyzers also trap ions but do so using a magnetic field. In this method, mass spectra are generated using the Fourier transform. FT-MS encompasses both FT ion cyclotron resonance (FTICR) and Orbitrap mass spectrometers, which are able to operate over a wide range of masses [35]. In addition to a wide mass range, these mass analyzers also have high sensitivity, mass accuracy, and resolution, though they tend also to be expensive and operationally complex [36,37,38,39]. TOF, quadrupole, ion trap, and FT-MS mass analyzers can be used separately but are often combined in order to exploit each instrument’s unique advantages. After passing through the mass analyzer, ions reach a detector according to their m/z value, and the corresponding data are ultimately generated as mass spectra.

### 2.5. Data Analysis

The analysis of MS/MS data involves assigning peptide sequences to the generated mass spectra, statistical scoring of the resultant peptide/spectrum matches, and identification of proteins from the filtered peptide list [40]. 

Multiple strategies exist for identification of peptides from mass spectra, but the predominant method is database searching, an approach that compares the experimental spectra to theoretical spectra generated via a protein sequence database. This approach calculates a series of search scores based on how closely the experimental and theoretical spectra match. Search parameters can be applied to aid in the identification of true versus false matches [40]. Multiple databases exist for this purpose, and the choice depends on the experimental goal [41]. RefSeq and Uniprot are among the most commonly used databases.

The database search strategy results in multiple candidate peptides ranked using the search score, with the most likely match typically being the top scoring candidate. However, more often than not, multiple spectra are analyzed at once, and in these cases a correction for multiple comparisons must be applied. Thus, the false discovery rate (FDR), which accounts for the expected amount of erroneous peptide/spectrum matches among the full set of matches, is typically used [40]. Use of a target–decoy database for calculating FDR allows estimation of false positives, while false negatives are estimated using multiple factors [7]. Quality control metrics are usually employed at the spectra or the peptide spectrum matching level. Depending on the goal of the project, metrics like the coefficient of variation, number of identifications, peptide types (i.e., tryptic status), charge state distribution, and the number of missed cleavages can be used to assess the mass spectrometry performance.

Following generation of a high-quality peptide list, proteins can be inferred. This step is often built into the databases used for peptide-to-spectrum matching. Other approaches include *de novo* sequencing, used when reference sequence is unavailable, or a combination of these techniques. Once a protein list is obtained, a multitude of tools exists for visualization and interpretation of the data as detailed in the following section.

In quantitative proteomics, the abundances of high-confidence proteins can be measured and compared across experimental samples. There are several different experimental approaches to measuring abundance, each with relative advantages across cost, complexity, sensitivity, range of proteins quantified, and instrument capability. For example, label-free quantification requires simple, inexpensive sample preparation and is supported on many instruments; however, it requires a separate instrument run for each sample and therefore requires careful normalization across samples. In contrast, isobaric tagging requires a more expensive sample preparation but yields more sensitive and accurate quantifications and is designed to multiplex several samples across a single run [40,41]. The most appropriate quantitation approach for a given experiment depends on the specifics of the research question.

Missing values are a common issue with proteomics studies, partially because of the nature of how mass spectrometers work. Although the most simplistic approach would be discarding any proteins that miss one or more measurements, the comparison of multiple experiments can prove to be quite challenging. Experiments containing peptides marked with isobaric tags, for example, can suffer from exponential increases in missing values depending on how many experiments are considered [42]. Data imputation is a common technique that might be used to mitigate such issues. Strategies can vary depending on the type of data and extension of the missingness rate.

Many tools exist for bioinformatic analyses of proteomic data, including general programs like Trans-Proteomic Pipeline [43] and MaxQuant [44], database search engines like Comet [45] and MSFragger [46], and post-processing tools like Philosopher [47] and TMT-Integrator (http://tmt-integrator.nesvilab.org/ accessed on 22 May 2021). A combination of several such tools is often necessary to infer biological meaning from the largescale datasets intrinsic to unbiased proteomic techniques.

### 2.6. Differential Protein Expression Analysis

Downstream statistical analysis of quantitative proteomic data can compare relative protein expression among experimental subgroups to identify distinguishing molecular expression profiles for a treatment group or phenotype. Techniques to detect a statistically significant difference in expression range from a straightforward *t*-test or analysis of variance (ANOVA) to more nuanced approaches that incorporate a linear model to mitigate the large variances common in smaller proteomics datasets [40,48,49].

Since proteins typically act in concert, it is often useful to place the differential expression results into a biological context using gene set enrichment and pathway analyses. Gene Ontology (GO) terms (http://geneontology.org accessed on 22 May 2021 [50]) are often assigned to the proteins in a sample as the first step in data interpretation. GO terms are hierarchically clustered and classified into three categories: biological processes, molecular functions, and cellular components. An enrichment analysis can be performed to show which GO terms are most abundant in a sample and to compare experimental groups or datasets [51]. Enrichment analysis, as well as several other types of annotation, including clustering GO terms, visualizing genes on pathway maps, and identifying interacting proteins, are often done with DAVID (https://david.ncifcrf.gov accessed on 22 May 2021), though many more tools expanding on GO are available. A popular resource for identifying protein–protein interactions is the STRING database (https://string-db.org accessed on 22 May 2021), which includes a literature-mining feature and has a GO classification built in. STRING identifies known physical and functional interactions as well as predicted interactions, and these data are easily imported to or queried from within Cytoscape, a popular data visualization tool [52]. Additional protein interaction databases include MINT (https://mint.bio.uniroma2.it accessed on 22 May 2021), BioGRID (https://thebiogrid.org accessed on 22 May 2021), IntAct (https://www.ebi.ac.uk/intact/ accessed on 22 May 2021), and PIPs (http://www.compbio.dundee.ac.uk/www-pips/ accessed on 22 May 2021) [51,53,54,55,56]. However, because STRING aggregates data from several of these databases, it is generally favored. 

Another popular bioinformatic tool for analysis of proteomic data is pathway analysis, which yields a more functional view of the proteins contained within a dataset. Several pathway databases exist, including KEGG (https://www.kegg.jp accessed on 22 May 2021), Reactome (https://reactome.org accessed on 22 May 2021), Ingenuity Pathway Knowledge Base, BioCarta (http://www.biocarta.com accessed on 22 May 2021), PANTHER (http://www.pantherdb.org accessed on 22 May 2021), and GenMAPP (http://www.genmapp.org accessed on 22 May 2021) [57,58,59,60,61]. This is by no means a comprehensive list; hundreds of other pathway databases exist. Many pathway analysis tools also incorporate GO annotation, interaction networks, and other features, providing a comprehensive set of analysis strategies. Ingenuity Pathway Analysis (Qiagen, Hilden, Germany) and iPathway Guide (Advaita, Ann Arbor, MI, USA) are two popular such tools.

### 2.7. Data Publication

Regarding data publication, there is much variation in the field of proteomics. For the experiments published in original research manuscripts, a portion or the full set of MS data is generally included as a supplementary material, though the proportion of the full dataset that is included and the format in which it appears vary from study to study. There is currently a push in the proteomics field for better and more coordinated sharing of data in order to facilitate the development of improved data analysis pipelines [62]. Several top proteomic journals mandate public sharing of data, typically providing authors various options for data deposition. The Proteomics Identification database, better known as PRIDE (https://www.ebi.ac.uk/pride accessed on 22 May 2021), is perhaps the most common resource for MS file sharing. PeptideAtlas (http://www.peptideatlas.org accessed on 22 May 2021) is another well-known repository and, along with PRIDE, was created as part of the ProteomeXchange consortium [63]. Both include data generated via both unbiased and targeted MS methods. Another data repository is Chorus (https://chorusproject.org accessed on 22 May 2021), which is searchable and allows MS file visualization [64]. The Global Proteome Machine Database (https://www.thegpm.org/ accessed on 22 May 2021) also provides these features but includes MS data from many species [65,66]. Recently, the Encyclopedia of Proteome Dynamics (https://peptracker.com accessed on 22 May 2021) was developed to address the lack of integration of data repositories with other online bioinformatic resources. This repository is connected to the STRING database to provide information on protein–protein interactions as well as to pathway and protein structure resources [62]. Though issues including searchability, integration with analysis tools, and user-friendliness have been addressed, a more coordinated effort for proteomic data sharing is still needed.

## 3. Prior Studies

Prior vitreous proteomic studies have employed a range of sample selection criteria, sample preparation protocols, and MS techniques, with widely varying results in terms of the number of proteins identified. Major MS-based proteomic studies of PDR vitreous are discussed below, in order from the greatest to the fewest number of proteins identified. The studies were identified via multiple PubMed searches and utilization of the authors’ own libraries. Proteomic studies utilizing only targeted techniques such as ELISA or other immunoassay were excluded from this review. Due to the large dataset size intrinsic to proteomic studies and the complexity of bioinformatic analyses, discussion of these studies is generally limited to the results deemed noteworthy by each study’s authors. Details regarding MS techniques and other experimental components are summarized in Table 1.

In a large-scale label-free MS study, Loukovaara et al. [67] analyzed 138 vitreous samples from diabetics with non-proliferative diabetic retinopathy (NPDR) and PDR, including patients treated with anti-VEGF prior to sample collection. They identified and quantified 2482 and 1351 proteins, respectively. Of the quantified proteins, they found 230 to be significantly more abundant in PDR than in NPDR. Differentially expressed proteins were found to belong to signaling pathways implicated in DR pathogenesis, including the complement and the coagulation pathways, cell adhesion molecules, and proteins involved in inflammation. Seventy-two proteins, including some belonging to these pathways, were found to be downregulated in PDR samples derived from the patients who had received anti-VEGF treatment. In this group of samples, 19 proteins were upregulated.

In a comparison of PDR patients undergoing vitrectomy for non-clearing vitreous hemorrhage (*n* = 5) and non-diabetic patients undergoing the same procedure for MH/ERM repair (*n* = 5), Gardner and Sundstrom identified 1213 and 929 proteins, respectively. Analysis of the PDR proteome revealed the presence of pathways mediating preclinical aspects of PDR pathogenesis (neuroprotection, oxidative stress), as well as clinical aspects of the disease (angiogenesis). In line with these results, analysis of pathway activation status demonstrated increased activation of vascular endothelial cell proliferation and decreased activation of neuronal and synaptic processes [19].

In a study comparing PDR samples from patients treated with anti-VEGF (*n* = 9) prior to vitrectomy, PDR samples from patients with no prior anti-VEGF treatment (*n* = 8), and MH samples from non-diabetic patients, Zou et al. identified 740 proteins in the combined PDR groups and 586 proteins in the MH group. Of the identified proteins, 307 were differentially expressed between treated and untreated PDR vitreous, 218 of which were downregulated in the treatment group. Bioinformatic analysis of differentially expressed proteins revealed involvement of the innate immune response, platelet degranulation, endocytosis, heme scavenging, and complement regulation, leading the authors to conclude that anti-VEGF treatment regulates the immune response [68].

Schori et al. [26] identified 677 unique proteins across vitreous samples from four patient groups: dry (*n* = 6) and neovascular (*n* = 10) age-related macular degeneration, PDR (*n* = 9), and ERM (*n* = 9). Among the top enriched proteins in the PDR group were hemoglobin subunit beta and carbonic anhydrase I, while the top pathways included complement- and micronutrient-related signaling.

Li et al. [69] analyzed vitreous samples from PDR (*n* = 9) and MH (*n* = 9), identifying 610 proteins, including 334 that were present in both groups and 62 that were differentially expressed by a fold change of > 2. Based on the bioinformatic analysis of the differentially expressed proteins, the authors concluded that the proteins involved in both immunity and transport may be implicated in PDR.

Using a variety of different MS techniques, Kim et al. analyzed vitreous samples from 11 patients with PDR and 14 patients with MH, resulting in the identification of 531 proteins. The authors highlighted groups of the proteins implicated in processes known to be associated with PDR pathogenesis, including several insulin-like growth factor (IGF) and IGF-binding proteins, mediators of angiogenesis and vascular permeability, and acute phase response proteins [70]. However, proteins were not quantified in this study, so the relative amounts of these proteins in PDR versus MH samples could not be assessed.

Gao et al. [71,72] performed two studies comparing the proteomes of the vitreous derived from patients with PDR, from patients with diabetes without clinically evident DR, and from non-diabetic patients undergoing repair of MH, ERM, or retinal detachment or receiving a glaucoma implant. These studies analyzed 17 and 25 samples, respectively, using similar MS methods. In a 2008 study, 252 proteins were identified, including 56 that were differentially expressed in diabetic versus non-diabetic samples. The authors highlighted the identification of 30 proteins involved in kinin–kallikrein, coagulation, and complement pathways, five of which were elevated in the PDR vitreous as compared to the non-diabetic vitreous. In a study from the year before, this group had identified 117 proteins and found that levels of carbonic anhydrase I were higher in the vitreous from patients with DR as compared to that of non-diabetic patients. The authors hypothesized that this finding indicates an influence of retinal hemorrhage and red blood cell lysis on the composition of the vitreous proteome in diabetic patients.

In an analysis of the vitreous from a single patient with PDR, Koyama et al. identified 84 unique proteins. Abundant plasma proteins were not depleted prior to MS, and several of the highest-abundance plasma proteins were detected in their analysis, so it is possible that less abundant proteins were masked. This was not a quantitative analysis, but the authors highlighted the presence of both proangiogenic (insulin-like growth factor, VEGF, fibroblast growth factor, placental endothelial cell growth factor) and antiangiogenic factors (pigment epithelium-derived growth factor (PEDF), endostatin, thrombospondin) [73].

Balaiya et al. [74] subjected each of the pooled PDR and MH/ERM samples derived from five patients to MS and detected 57 proteins, 16 of which were unique to the PDR vitreous. The identified proteins included several components of the complement cascade, acute phase reactants, transport proteins, and a small number of proteins involved in the visual cycle. Many of the detected proteins were plasma proteins, so the relatively low number of proteins identified could have resulted from a lack of abundant protein depletion.

In a study of 33 PDR and 26 MH vitreous samples, Yamane et al. [75] identified 38 unique proteins. Many of the identified proteins in this study are also abundantly present in plasma, and the majority of the identified proteins were present in corresponding serum samples. A portion of the identified plasma proteins were increased in the PDR vitreous relative to the MH vitreous, so the authors asserted that this difference indicated increased retinal vascular permeability in PDR. Depletion of abundant proteins prior to MS analysis likely would have unmasked less abundant proteins in this study. 

Wang et al. [76] identified 29 proteins in a study of 10 PDR and 10 corneal transplant vitreous samples. In contrast to other studies discussed here, the majority of the identified proteins were present in decreased amounts in PDR samples. Among the downregulated proteins was clusterin, which plays a role in protection against blood–retinal barrier breakdown. Other differentially expressed proteins identified in this study are thought to be involved in neovascularization, endothelial dysfunction, and cell cycle progression.

Using differential gel electrophoreses (DIGE) followed by MS in an analysis of the PDR (*n* = 4), diabetic macular edema (*n* = 4), and MH (*n* = 8) vitreous, Hernandez et al. [28] identified 25 proteins from 1300 spots, 81 of which were differentially expressed between two groups. Six proteins (beta 2-glycoprotein, gelsolin, retinol-binding protein 3, metalloproteinase inhibitor 2, prostaglandin-H2, D-isomerase, and vitamin D-binding protein) were found to be related to PDR. Similar to other studies, many complement components and other abundant plasma proteins were also identified.

In a study pairing two-dimensional gel electrophoresis (2-DE) with MS, Kim et al. [77] identified 23 proteins using 15 PDR and 15 MH vitreous samples. Eight of these 23 proteins were differentially expressed between the experimental groups. Note that while protein spots from 2-DE were quantified according to relative intensities across the experimental groups, MS analysis was nonquantitative. The proteins found to be upregulated in PDR relative to the controls included PEDF, serine protease inhibitor, prostaglandin-H2 D-isomerase, apolipoprotein A-IV precursor, and alpha-2-HS-glycoprotein. In contrast, α_1_-antitrypsin precursor, ankyrin repeat domain 15 protein, and beta V spectrin were downregulated in PDR.

García-Ramírez et al. [78] performed MS analysis of eight PDR and 10 MH vitreous samples, resulting in the identification of 11 proteins, eight of which were increased in the PDR vitreous as compared to the MH vitreous. As the only two abundant proteins, albumin and IgG, were depleted prior to the analysis, several of the 11 identified proteins were abundant plasma proteins. Based on the elevated portion of proteins, the authors suggested a role for inflammation and the complement cascade in PDR. Among the proteins expressed in lower amounts in the PDR vitreous were PEDF, which has neuroprotective and antiangiogenic properties, and the interphotoreceptor retinoid-binding protein, which plays a role in the visual cycle.

### 3.1. Major Findings

The biological significance of the findings in the studies discussed above was subject to the original authors’ interpretation and is reported here according to what those authors chose to highlight. Generally, authors focused on the proteins and pathways related to the pathogenesis of PDR or those that had not been identified in prior studies. In most cases, authors focused on those proteins that were upregulated in the PDR vitreous, though consideration of the proteins that are decreased in these samples may also prove worthwhile.

Upregulations in complement-, inflammation-, and angiogenesis-related molecules were among the most frequently highlighted findings in the PDR vitreous. Loukovaara et al. highlighted the elevated complement components as an important therapeutic target, as biologics targeting these molecules are already in development for AMD [67]. Gardner and Sundstrom highlighted the presence and activation of angiogenic pathways and the simultaneous deactivation of neuronal pathways in the PDR vitreous, emphasizing a comprehensive view of PDR pathogenesis, focusing on both early neurodegenerative changes and later-stage vascular abnormalities [19]. Comparing differential expression in the vitreous from patients who received intravitreal anti-VEGF prior to sample collection to their treatment-naïve counterparts, Zou et al. suggested that this treatment has effects across multiple signaling pathways rather than solely on the VEGF molecule itself [68]. Schori et al. stressed the differences in pathways identified in PDR versus AMD vitreous, noting enrichment in the complement- and coagulation-related proteins in PDR [26]. Li et al. focused on the identification of six apolipoproteins in PDR vitreous as well as on the molecules known to regulate blood pressure, inflammation, and vascular permeability [69]. Kim et al. and Yamane et al. both emphasized the presence of proteins related to vascular permeability [70,75]. Similarly, Gao et al. cited the presence of red blood cell proteins as an indicator of retinal hemorrhage and red blood cell lysis in PDR eyes [71,72]. Koyama et al. noted the presence of both pro-and antiangiogenic factors in PDR vitreous but did not quantify them [73]. Balaiya et al. highlighted an upregulation of proteins that take part in coagulation, complement, and kinin–kallikrein pathways in PDR vitreous, along with an absence of a protein involved in the visual cycle, potentially representing a neurodegenerative change [74]. Similarly, Hernandez et al. also identified numerous complement pathway components [28]. Wang et al. highlighted the downregulation of the molecule that helps maintain the blood–retinal barrier and the presence of the proteins involved in neovascularization [76]. Kim et al. noted an upregulation of the antiangiogenic molecule PEDF in the PDR vitreous, concluding that PEDF is only one of multiple modulators of angiogenesis implicated in PDR. Additionally, this study found decreased expression of the molecules associated with photoreceptor outer segments and lipid metabolism, hypothesizing that these changes may represent components of PDR pathogenesis [77]. García-Ramirez, similarly to the other studies reviewed here, noted an increase in complement components in the PDR vitreous. PEDF and a visual cycle protein were both downregulated in the PDR vitreous, reflecting the angiogenic and neurodegenerative changes that characterize the disease [78].

### 3.2. Shortcomings

A major shortcoming of a subset of the discussed studies is limited coverage of the total vitreous proteome. Due to the variation in sample preparation and MS techniques utilized, the number of proteins identified in the highlighted studies ranged from 11 to 2482. Although the total number of the proteins constituting the vitreous proteome is unknown, those studies that measured fewer than approximately 1200 proteins covered less than half of the vitreous proteome that is measurable using current technologies. In some cases, the limited number of identified proteins was likely due to a lack of depletion of abundant plasma proteins during sample preparation, leading to masking of less abundant components of the vitreous proteome. In other cases, the protein fractionation technique was likely a limiting factor. For example, studies using DIGE generally identified fewer proteins. The effect of various steps of sample preparation and MS instrumentation on the number of proteins able to be identified is discussed in more detail in Section 2.

Another frequent shortcoming of vitreous proteomic studies is overinterpretation of bioinformatic analyses. Pathway and enrichment analyses are critically useful in placing patterns of differential protein expression into a biological context. However, these analyses often produce a large number of false-positive associations. With this in mind, we recommend transforming candidate pathway/enrichment significance scores with a false discovery rate adjustment (e.g., using the Benjamini–Hochberg procedure). Furthermore, since proteins function in pathways which have upregulating and downregulating components, pathway/enrichment analyses that consider either the pathway activation/inhibition topology or coherence of up- and downregulation across sample groups will focus the biological results on a subset of candidate biological pathways/protein sets. Finally, it is critical to use the most current gene set/pathway definitions when executing these analyses [79,80,81]. 

Lastly, data sharing across these studies has been inconsistent and insufficient. Only two of the above studies deposited the full MS dataset in a public repository. Several studies included the complete dataset or a portion of data in the manuscript or accompanying supplementary material. Others, however, did not publish the full dataset at all. Even when datasets are shared, the format varies from study to study, making it cumbersome to interpret and compare datasets. Requirements for data formatting vary from journal to journal but generally cover details pertaining to experimental design, search parameters, protein and peptide identification details, post-translational modifications, protein interference information, and quantitative measurements. Submission of raw MS data may also be mandated.

## 4. Moving the Field Forward

Vitreous proteomic studies have provided insight into the pathogenesis of PDR and may represent an opportunity for discovery of novel disease biomarkers and therapeutic targets. In order to succeed in these endeavors, researchers performing these studies should address the shortcomings discussed above in the following ways.

First, quantitative proteomics experiments are vulnerable to inconsistent quantification across samples (i.e., protein dropout) and large variance in measured protein abundance. These characteristics underscore the importance of considering both type I (false-positive) and type II (false negative) errors in the initial experimental/analytical design. The proportions of these errors are an attribute of an individual protein and are determined by the protein’s abundance distributions across experimental groups. Therefore, when considering an individual protein, to confidently reject or fail to reject a putative difference among experimental groups, it is imperative that one has a sufficient sample size to faithfully represent those distributions. One of the discussed studies used only a single vitreous sample, another used 74 samples in their experimental group, and the sample size in other studies varied across that range. With this in mind, untargeted quantitative proteomics experiments should publish all the measured protein abundances along with the calculated effect sizes so that the follow-on experiment designs can leverage these data to tune the sample size for proteins of interest. Note that thresholds of significance (probability of false positive) and power (1—probability of false negatives) are commonly set at 0.05 and 0.8, respectively. However, because the cost of false positives and false negatives is specific to an experiment, these cutoffs are notional and should be informed by the research question.

Second, a method for normalization would be beneficial in order to enable comparison analyses across separate datasets. This would be accomplished in the form of identical samples being included in MS experiments performed at separate timepoints, which has yet to be included in a published PDR vitreous proteomics experiment.

Lastly, the full dataset generated by published vitreous proteomic studies should always be deposited in a freely accessible public repository, such as PRIDE (https://www.ebi.ac.uk/pride/ accessed on 22 May 2021) or Peptide Atlas (http://www.peptideatlas.org accessed on 22 May 2021). Care should be taken to publish these data in a format that will be easily interpreted by outside researchers. Better access to data from other groups would facilitate collaboration and improve the context in which new studies can be interpreted. Ultimately, these changes could facilitate new discoveries and a better understanding of PDR.

## Figures and Tables

**Table 1:** Summary of prior mass spectrometry-based proteomic studies of a proliferative diabetic retinopathy vitreous. Experimental details of the studies described in the text are outlined here in order from the highest to the lowest number of proteins measured.

Publication	PMID	Experimental Groups	Total Number of Samples	Sample Size by Group	Mass Spectrometry	Instrument	Proteins Measured	Repository
Loukovaara, S., et al. Quantitative Proteomics Analysis of Vitreous Humor from Diabetic Retinopathy Patients. *J. Proteome Res.* **2015**, *14 (12)*, 5131–5143.	26490944	PDR/NPDR/PDR (anti-VEGF)/NPDR (anti-VEGF)	138	74 PDR, 49 NPDR, 5 PDR (anti-VEGF), 10 NPDR (anti-VEGF)	LC-MS/MS	Orbitrap Elite	2482	Peptide Atlas
Gardner, T.W., and Sundstrom, J.M. A proposal for early and personalized treatment of diabetic retinopathy based on clinical pathophysiology and molecular phenotyping. *Vision Res.* **2017**.	28438679	PDR + NCVH/MH/ERM	10	5 PDR + NCVH, 5 MH/ERM	Nano-LC-MS/MS	Q Exactive	1213	Supplemental
Zou, C., et al. Difference in the Vitreal Protein Profiles of Patients with Proliferative Diabetic Retinopathy with and without Intravitreal Conbercept Injection. *J. Ophthalmol.* **2018**, 7397610.	29850212	PDR + IVC/PDR (no IVC)/MH	26	9 PDR + IVC, 8 PDR (no IVC), 9 MH	LC-MS/MS	Not listed	740	Supplemental
Schori, C., et al. The Proteomic Landscape in the Vitreous of Patients with Age-Related and Diabetic Retinal Disease. *Investig. Ophthalmol. Vis. Sci.* **2018**, *59 (4)*, AMD31–AMD40.	30025106	PDR/dry AMD/NV AMD/ERM	34	9 PDR, 6 dry AMD, 10 NV AMD, 9 ERM	Nano-LC-MS/MS	Orbitrap Fusion	677	PRIDE
Li, J., et al. Quantitative proteomics analysis of vitreous body from type 2 diabetic patients with proliferative diabetic retinopathy. *BMC Ophthalmol.* **2018**, *18 (1)*, 151.	29940965	PDR/MH	18	9 PDR, 9 MH	LC-MS/MS	Orbitrap Elite	610	Full dataset not included
Kim, T., et al. Profiling of vitreous proteomes from proliferative diabetic retinopathy and nondiabetic patients. *Proteomics* **2007**, *7 (22)*, 4203–4215.	17955474	PDR/MH	33	11 PDR, 14 MH	IS/2-DE/MALDI-MS, nano-LC-MALDI-MS/MS, nano-LC-ESI-MS/MS	Thermo Electron model LTQ ESI linear single-quadrupole IT	531	Supplemental
Gao, B.-B., et al. Characterization of the Vitreous Proteome in Diabetes without Diabetic Retinopathy and Diabetes with Proliferative Diabetic Retinopathy. *J. Proteome Res.* **2008**, *7*, 2516–2525.	18433156	PDR/diabetic (no DR)/not diabetic	17	7 PDR, 4 diabetic (no DR), 6 not diabetic	Nano-LC-MS/MS	LTQ Linear Ion Trap	252	Supplemental
Gao, B.-B., et al. Extracellular carbonic anhydrase mediates hemorrhagic retinal and cerebral vascular permeability through prekallikrein activation. *Nat. Med.* **2007**, *13 (2)*, 181–188.	17259996	PDR/diabetic (no DR)/not diabetic	25	13 PDR, 4 diabetic (no DR), 8 not diabetic	MS/MS	LTQ Linear Ion Trap	117	Supplemental
Koyama, R., et al. Catalogue of soluble proteins in human vitreous humor by one-dimensional sodium dodecyl sulfate-polyacrylamide gel electrophoresis and electrospray ionization mass spectrometry including seven angiogenesis-regulating factors. *J. Chromatogr. B Analyt. Technol. Biomed. Life Sci.* **2003**, *792 (1)*, 5–21.	12828993	PDR	1	1 PDR	ESI-IT-MS/MS	LCQ^DECA^	84	Manuscript
Balaiya, S., et al. Characterization of Vitreous and Aqueous Proteome in Humans With Proliferative Diabetic Retinopathy and Its Clinical Correlation. *Proteomics Insights* **2017**, *8*, 1178641816686078.	28469465	PDR/MH/ERM	10	5 PDR, 5 MH/ERM	Nano-LC-MS/MS	LTQ Orbitrap XL	57	Manuscript, supplemental
Yamane, K., et al. Proteome analysis of human vitreous proteins. *Mol. Cell. Proteomics* **2003**, *2 (11)*, 1177–1187.	12975481	PDR/MH	59	33 PDR, 26 MH	ESI-MS, MALDI-MS	Q-TOF, Voyager-DE STR	38	Manuscript
Wang, H., et al. Characterisation of the vitreous proteome in proliferative diabetic retinopathy. *Proteome Sci.* **2012**, *10 (1)*, 15.	22390717	PDR/corneal transplant	20	10 PDR, 10 corneal transplants	DIGE + MALDI-MS	Not listed	29	Full dataset not included
Hernandez, C., et al. Identification of new pathogenic candidates for diabetic macular edema using fluorescence-based difference gel electrophoresis analysis. *Diabetes Metab. Res. Rev.* **2013**, *29 (6)*, 499–506.	23568601	PDR/DME/MH	16	4 PDR, 4 DME, 8 MH	DIGE + MALDI-MS	Ultraflex	25	Full dataset not included
Kim, S.J., et al. Differential expression of vitreous proteins in proliferative diabetic retinopathy. *Curr. Eye Res.* **2006**, *31 (3)*, 231–240.	16531280	PDR/MH	30	15 PDR, 15 MH	2-DE + MALDI-TOF, 2-DE + MS/MS	Not listed	23	Manuscript
Garcia-Ramirez, M., et al. Proteomic analysis of human vitreous fluid by fluorescence-based difference gel electrophoresis (DIGE): a new strategy for identifying potential candidates in the pathogenesis of proliferative diabetic retinopathy. *Diabetologia* **2007**, *50 (6)*, 1294–1303.	17380318	PDR/MH	18	8 PDR, 10 MH	DIGE + MALDI-MS	Ultraflex	11	Manuscript

Abbreviations: 2-DE, two-dimensional gel electrophoresis; AMD, age-related macular degeneration; DIGE, difference gel electrophoresis; DME, diabetic macular edema; DE, delayed extraction; DR, diabetic retinopathy; ERM, epiretinal membrane; ESI, electrospray ionization; IS, immunoaffinity subtraction; IT, ion trap; IVC, intravitreal conbercept; LC, liquid chromatography; LCQ^DECA^, trademarked name of a mass spectrometer (not an abbreviation); LTQ, linear trap quadrupole; MALDI, matrix-assisted laser desorption/ionization; MH, macular hole; MS, mass spectrometry; NCVH, non-clearing vitreous hemorrhage; NPDR, non-proliferative diabetic retinopathy; PDR, proliferative diabetic retinopathy; STR, short tandem repeat; TOF, time of flight; VEGF, vascular endothelial growth factor.
